# Green Synthesis of InP/ZnS Core/Shell Quantum Dots for Application in Heavy-Metal-Free Light-Emitting Diodes

**DOI:** 10.1186/s11671-017-2307-2

**Published:** 2017-09-19

**Authors:** Tsung-Rong Kuo, Shih-Ting Hung, Yen-Ting Lin, Tzu-Lin Chou, Ming-Cheng Kuo, Ya-Pei Kuo, Chia-Chun Chen

**Affiliations:** 1 Graduate Institute of Nanomedicine and Medical Engineering, College of Biomedical Engineering, Taipei Medical University, Taipei, 11031 Taiwan; 2 International Ph.D. Program in Biomedical Engineering, College of Biomedical Engineering, Taipei Medical University, Taipei, 11031 Taiwan; 3 Department of Chemistry, National Taiwan Normal University, Taipei, 11677 Taiwan; 4 AU Optronics Corporation, New Display Process Division, HsinChu, 30078 Taiwan

**Keywords:** InP/ZnS core/shell QDs, Light-emitting diodes, Heavy-metal-free, Environment-friendly, Green synthesis

## Abstract

Quantum dot light-emitting diodes (QD-LEDs) have been considered as potential display technologies with the characterizations of high color purity, flexibility, transparency, and cost efficiency. For the practical applications, the development of heavy-metal-free QD-LEDs from environment-friendly materials is the most important issue to reduce the impacts on human health and environmental pollution. In this work, heavy-metal-free InP/ZnS core/shell QDs with different fluorescence were prepared by green synthesis method with low cost, safe, and environment-friendly precursors. The InP/ZnS core/shell QDs with maximum fluorescence peak at ~ 530 nm, superior fluorescence quantum yield of 60.1%, and full width at half maximum of 55 nm were applied as an emission layer to fabricate multilayered QD-LEDs. The multilayered InP/ZnS core/shell QD-LEDs showed the turn-on voltage at ~ 5 V, the highest luminance (160 cd/m^2^) at 12 V, and the external quantum efficiency of 0.223% at 6.7 V. Overall, the multilayered InP/ZnS core/shell QD-LEDs reveal potential to be the heavy-metal-free QD-LEDs for future display applications.

**Electronic supplementary material:**

The online version of this article (10.1186/s11671-017-2307-2) contains supplementary material, which is available to authorized users.

## Background

With unique physical and chemical properties, quantum dots (QDs) have attracted great interest in applications such as lasers, biomedical imaging, sensors, and light-emitting diodes (LEDs) [1–9]. The QDs have been actively investigated for LED applications because of their attractive properties of size-tunable band gaps, good photostability, superior photoluminescence efficiency, and compatibility with solution-processing methods. The QD-LEDs have been considered as potential display technologies with the characterizations of high color purity, flexibility, transparency, and cost efficiency [10–16]. Currently, most of QD-LEDs have been manufactured by cadmium-based QDs, which are proved relatively easy to synthesize with high-quality optical properties [17]. However, the heavy-metal nature of the cadmium-based QDs has raised many concerns about carcinogenicity and other chronic health risks as well as disposal hazards. The regulatory acceptance of any heavy-metal compositions in QDs will severely obstruct the final commercialization of the QD-LEDs products. For the practical applications, the development of heavy-metal-free QD-LEDs is the most important issue to reduce the impacts on human health and environmental pollution.

To date, to eliminate the healthy and environmental concerns, many efforts have been focused on the syntheses of cadmium-free QDs for LED applications [18–24]. In recent studies, red emission of ZnCuInS/ZnS core/shell QDs mixed with blue-green emission of poly(*N*,*N*′-bis(4-butylphenyl)-*N*,*N*′-bis(phenyl)benzidine) have been applied to obtain white electroluminescence LEDs [25]. Highly stable and luminescent InP/GaP/ZnS core/shell/shell QDs with a maximum quantum yield of 85% have been used to fabricate white QD-LEDs with luminous efficiency of 54.71 lm/W, Ra of 80.56, and correlated color temperature of 7864 K at the color coordinate (0.3034, 0.2881) [26]. White QD-LEDs based on high-quality InP/ZnS core/shell QDs with luminescence tunable over the entire visible spectrum have been demonstrated with a high color rendering index of 91 [27]. Among these materials, indium phosphide (band gap ~ 1.35 eV) with core/shell structure is the potential candidate as the ideal alternative material to provide the similar emission wavelength range without intrinsic toxicity in comparison to cadmium-based QDs. Many studies have reported the synthetic approaches such as hot-injection, solvothermal, and heating-up method to synthesize InP/ZnS core/shell QDs with high quantum efficiency [28–30]. Several phosphorus precursors including tris(trimethylsilyl)phosphine, triarylsilylphosphines, phosphine, P_4,_ and PCl_3_ have been respectively utilized for the syntheses of InP/ZnS core/shell QDs [31–38]. However, these phosphorus precursors exhibiting some disadvantages such as expensive, flammable, and toxic have inhibited the further production of InP/ZnS core/shell QDs. Therefore, the green synthesis of InP/ZnS core/shell QDs by cheap, safe, and environment-friendly precursors is still the challenge in the field of materials science. Moreover, the use of InP/ZnS core/shell QDs to fabricate highly efficient QD-LEDs is also an important issue for practice application in display technology.

Herein, environment-friendly InP/ZnS core/shell QDs were successfully synthesized by solvothermal green synthesis with low-cost and safe precursors including InI_3_, ZnCl_2_, (DMA)_3_P, zinc stearate, and sulfur. The structural and optical properties of InP/ZnS core/shell QDs were characterized by transmission electron microscopy (TEM), powder X-ray diffraction (XRD), and ultraviolet-visible (UV-Vis) spectrophotometer. Thermal stability of the fluorescence of InP/ZnS core/shell QDs was investigated to find the optimal process temperature for further fabrication of multilayered InP/ZnS core/shell QD-LEDs. Moreover, the performance of multilayered InP/ZnS core/shell QD-LEDs was explored to demonstrate the possibility for the practical applications such as displays in the near future.

## Methods

### Chemicals

Indium (III) iodide (InI_3_), zinc (II) chloride (ZnCl_2_), tris(dimethylamino)phosphine (DMA)_3_P, and zinc stearate were purchased from Sigma-Aldrich. Oleylamine was purchased from Acros Organics. Trioctylphosphine (TOP) and sulfur powder were purchased from Strem Chemicals. Octadecene (ODE) was purchased from Alfa Aesar.

### Preparation of InP/ZnS Core/Shell QDs

InP/ZnS core/shell QDs were synthesized via solvothermal green synthesis according to the previous study with some modifications [39]. First, 224 mg of InI_3_, 300 mg of ZnCl_2_, and 5.0 mL of oleylamine were added into a three-neck round-bottom flask. The reactants were stirred and degassed at 120 °C for 60 min and then heated to 180 °C under argon atmosphere. At 180 °C, 0.45 mL of (DMA)_3_P was quickly injected into the above reactants. After the phosphorus precursor injection, the InP QDs were continually grown for 20 min. Second, for the growth of ZnS shell onto InP core, 1.5 g of zinc stearate and 6 mL ODE were mixed as zinc precursor. In addition, 0.72 g sulfur and 10 mL TOP were mixed as sulfur precursor. To synthesize InP/ZnS core/shell QDs, 1 mL of sulfur precursor was slowly injected into the InP QDs solution at 180 °C. At 40 min after the injection of sulfur precursor, the solution with InP QDs and sulfur precursor was heated to 200 °C and then the solution was added with 4 mL of zinc precursor. After 60 min, the solution with InP QDs, sulfur precursor, and zinc precursor was heated at 220 °C for 30 min to allow the growth of ZnS shell onto InP core. Afterward, the additional sulfur precursor (0.7 mL) was added to the solution with InP/ZnS core/shell QDs for second growth of the ZnS shell. After second injection of sulfur precursor, the solution was heated to 240 °C and kept at 240 °C for 30 min. After 30 min, the zinc precursor (2 mL) was added to the solution with InP/ZnS core/shell QDs and second injection sulfur precursor. After second injection of zinc precursor, the solution was heated to 260 °C to continue growth for 30 min. For the preparations of red and yellow fluorescent InP/ZnS core/shell QDs, the indium precursors of InCl_3_ and InBr_3_ were respectively used to synthesize red and yellow fluorescent InP/ZnS core/shell QDs. After the synthetic processes, the solution of InP/ZnS core/shell QDs was cooled down to room temperature. To remove the unreacted compounds and byproducts, the solution of InP/ZnS core/shell QDs was washed with small amount of acetone and then centrifuged at 4000 rpm for 15 min. After centrifugation, the supernatant was carefully removed without disturbance. The precipitate was redispersed in the solvent composed by chloroform and acetone (20/80, *v*/*v*) and then centrifuged at 4000 rpm for 15 min. After removal of supernatant, the InP/ZnS core/shell QDs were dispersed in chloroform for further QD-LED applications.

### Thermal Stability Test of InP/ZnS Core/Shell QDs

To test the thermal stability, the InP/ZnS Core/Shell QD solution was first deposited by spin casting (1500 rpm, 60 s) on the glass slides. And then the glass slides coated with InP/ZnS core/shell QDs were respectively annealed under temperatures including 25, 70, 100, 130, and 150 °C. After annealing with different times, the fluorescence of the glass slides coated with InP/ZnS core/shell QDs was measured by gel/fluorescence/chemiluminescence imaging system. The changes of fluorescence of the glass slides coated with InP/ZnS core/shell QDs were calculated by ImageJ software.

### Materials Characterizations

A Philips Technai G2 transmission electron microscopy (TEM) was operated at 200 kV to acquire TEM images. To prepare TEM samples, the InP/ZnS core/shell QDs were ultrasonically dispersed in chloroform and then a drop of the InP/ZnS core/shell QD solution was casted off onto a copper-carbon TEM grid. Subsequently, the resulting TEM grid was dried in air. X-ray diffraction (XRD) measurements were obtained by Bruker D8 tools advance, operating with Cu Kα radiation (λ = 1.5406 Å) generated at 40 keV and 40 mA. UV-Vis absorption spectra were measured by V-770ST UV/Vis spectrophotometer. Fluorescence spectra were obtained by SLM Aminco-Bowman Series 2.

### Fabrication of Multilayered InP/ZnS Core/Shell QD-LEDs

Multilayered InP/ZnS core/shell QD-LEDs were fabricated via sequential depositions of the constituent layers including hole injection layer (HIL), hole transport layer (HTL), emitting layer (InP/ZnS core/shell QDs, EML), exciton block layer (EBL), electron transport layer (ETL), and electron injection layer (EIL) on the substrate of AU Optro﻿nics (AUO) normal bottom emission (BE) model test (MT﻿). The constituent layers of HIL, HTL, EBL, ETL, EIL, and substrate of AUO normal BE MT were provided by AU Optronics Corporation. For the fabrication of multilayered InP/ZnS core/shell QD-LEDs, the layers of HIL, HTL, and EML were sequentially deposited by spin casting on the substrate of AUO normal BE MT. The solution concentration of InP/ZnS core/shell QDs was 20 mg/mL. The solution of InP/ZnS core/shell QDs (20 mg/mL) was spin casted (1500 rpm) to form the EML. Afterward, to dry the EML, the substrate of AUO normal BE MT with HIL, HTL, and EML was baked at 70 °C. Finally, the layers of EBL, ETL, EIL, and Al cathode were sequentially deposited on EML by thermal vapor deposition. The light-emitting area of the multilayered InP/ZnS core/shell QD-LEDs was 0.2 × 0.2 cm^2^. The film thickness of multilayered InP/ZnS core/shell QD-LEDs was measured by the α-step apparatus. The performance of multilayered InP/ZnS core/shell QD-LEDs was detected by PR670 photometers (Titan Electro-Optics Co., Ltd).

## Results and Discussion

### Characterizations of InP/ZnS Core/Shell QDs

**Fig. 1:**
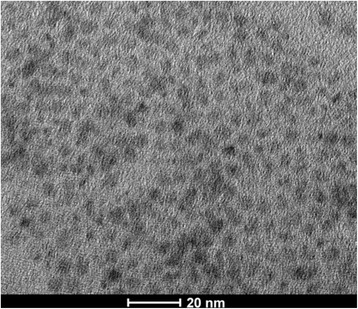
TEM image of InP/ZnS core/shell QDs

InP/ZnS core/shell QDs were prepared by solvothermal green synthesis with cheap, safer, and environment-friendly precursors including InI_3_, ZnCl_2_, (DMA)_3_P, zinc stearate, and sulfur compared to previous studies. In previous work, ZnCl_2_ has been demonstrated to facilitate the ZnS shell growth and to reduce the size distribution of the InP core [39]. For the formation of InP core, the phosphorus precursor of (DMA)_3_P was used because of its low price. More importantly, the (DMA)_3_P is stable under ambient conditions for the improvement of the safety of InP synthesis. As shown in the TEM image of Fig. 1, the InP/ZnS core/shell QDs revealed the spherical morphology. After statistics of 100 QDs in the TEM image, the average diameter of InP/ZnS core/shell QDs was ~ 4 nm. Histogram of size distribution of InP/ZnS core/shell QDs and Gaussian fitting were shown in the Additional file 1: Figure S1. The EDX analysis of InP/ZnS core/shell QDs showed that the atomic percentages of phosphorus, sulfur, zinc, and indium were respectively 21.20, 4.17, 69.27, and 5.36% as shown in Additional file 1: Figure S2.

**Fig. 2:**
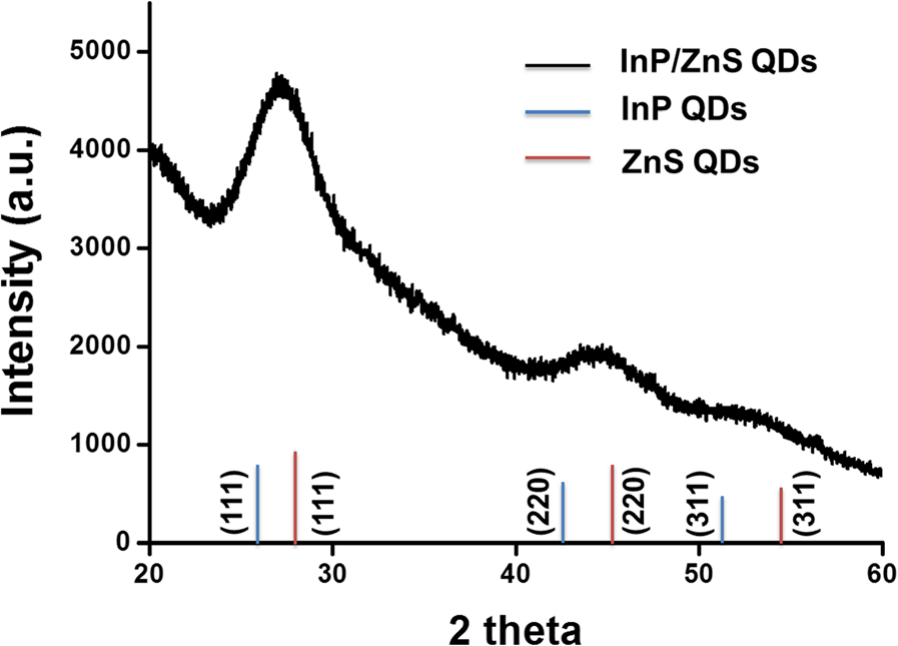
XRD patterns of InP/ZnS core/shell QDs. The XRD reflection peaks of InP QDs and ZnS QDs with typical zinc-blend phase

To confirm the structure of InP/ZnS core/shell QDs, the X-ray diffraction (XRD) pattern was examined (Fig. 2). The main peaks of InP QDs (JCPDS 73-1983) at 26.3°, 43.6°, and 51.6° were indexed to the (111), (220), and (311) planes of zinc blende structure, respectively. The peaks located at 28.5°, 47.4°, and 56.3° were respectively responded to the (111), (220), and (311) planes of zinc blende structure (JCPDS 77-2100) for ZnS. The XRD pattern showed that the diffraction peaks of InP and ZnS shifted to the positions between their theoretical values in the InP/ZnS core/shell QDs. The reason was attributed to the lattice mismatch between InP and ZnS as demonstrated before for CdSe/CdS core/shell QDs [40]. As shown in the XRD pattern, the lattice mismatch also revealed that the InP/ZnS core/shell QDs were successfully obtained by solvothermal green synthesis with the cheap, safe, and environment-friendly precursors.

**Fig. 3:**
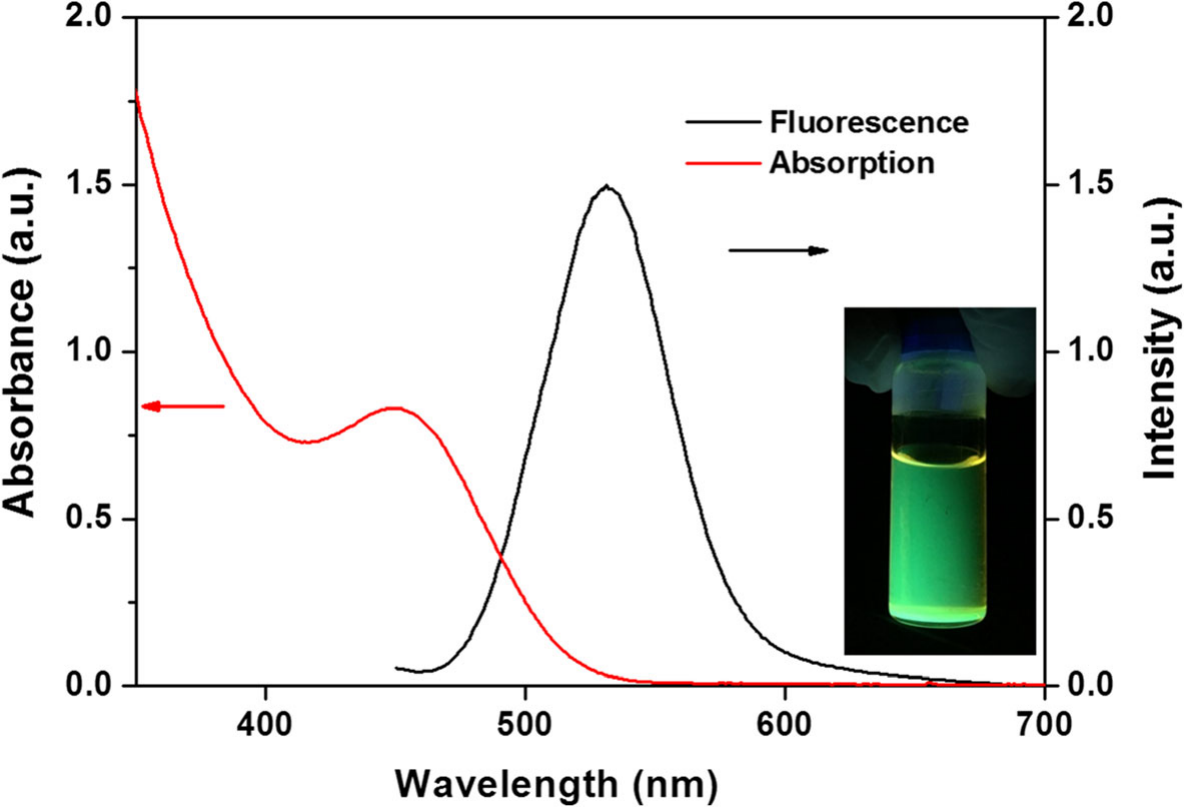
UV-Vis spectra (red line) and fluorescence spectra (black line) of InP/ZnS core/shell QDs. The inset showed the green fluorescence of InP/ZnS core/shell QDs irradiated by hand-held long-wave UV lamp

To further investigate the optical properties, (UV-Vis) spectra and fluorescence spectra of InP/ZnS core/shell QDs were measured before the fabrication of QD-LEDs. In Fig. 3, the absorption peak of InP/ZnS core/shell QDs was located at ~ 480 nm. The maximum fluorescence emission peak of InP/ZnS core/shell QDs was obtained at ~ 530 nm. In the fluorescence spectra, the full width at half maximum of InP/ZnS core/shell QDs was calculated to be ~ 55 nm. The fluorescence quantum yield of InP/ZnS core/shell QDs was estimated to be 60.1% in comparison with fluorescein (see Additional file 1 for the calculation of fluorescence quantum yield). The inset in Fig. 3 showed the green fluorescence of InP/ZnS core/shell QDs with the irradiation by hand-held long-wave UV lamp. The excellent optical properties of InP/ZnS core/shell QDs are suitable for the fabrication of green QD-LEDs. Furthermore, the InP/ZnS core/shell QDs with red and yellow fluorescence were also successfully prepared by the solvothermal green synthesis as shown in the Additional file 1: Figure S3.

### Performance of InP/ZnS Core/Shell QD-LEDs

**Fig. 4:**
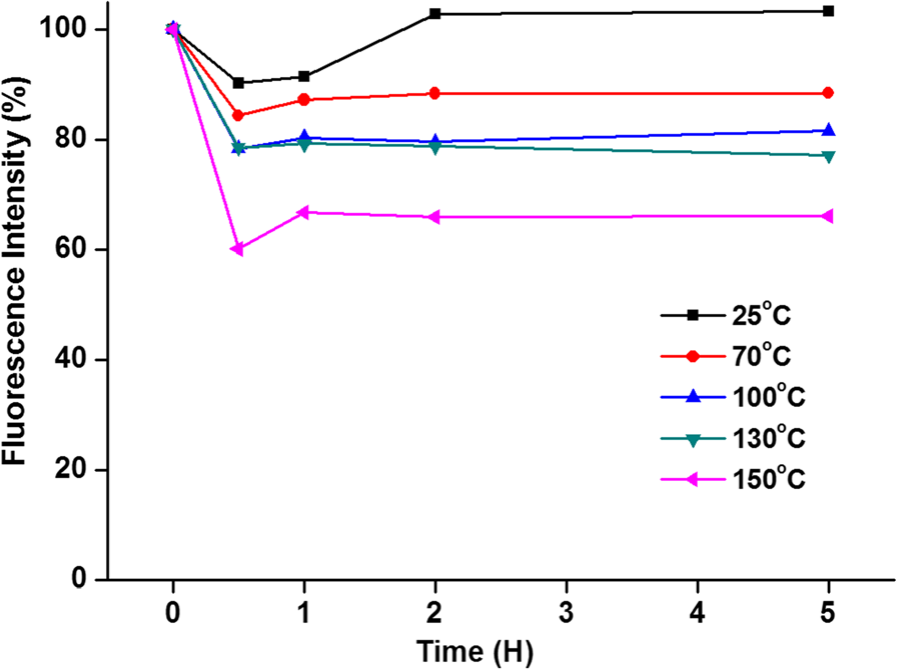
The changes of fluorescence intensities of InP/ZnS core/shell QDs with different annealing temperatures

Thermal stability of the fluorescence of InP/ZnS core/shell QDs is an important factor for the fabrication and performance of QD-LEDs. To investigate the thermal stability of fluorescence, the InP/ZnS core/shell QDs were annealed under different temperatures. As shown in Fig. 4, the fluorescent intensities of InP/ZnS core/shell QDs were decreased with annealing temperatures from 25 to 150 °C in the first hour. Previous studies have demonstrated the decrease of fluorescence of QDs as the increase of temperature [41–43]. However, the fluorescent intensities of InP/ZnS core/shell QDs were slightly increased after annealing for 1 hour. The simple annealing process diminished the accumulated defect states within the InP/ZnS core/shell QDs and therefore decreased the non-radiative recombination [44]. Although the fluorescence intensity of InP/ZnS core/shell QDs showed no significant change with annealing temperature under 25 °C, the annealing temperature of 25 °C was not suitable for the fabrication of QD-LEDs. During the QD-LED preparation, the minimal process temperature is 70 °C because the QD-LEDs need to be baked above 70 °C to dry the devices. As shown in Fig. 4, after 5 h annealing, the fluorescence intensities of InP/ZnS core/shell QDs with annealing temperatures of 70, 100, 130, and 150 °C were respectively maintained at 88, 81, 77, and 66% in comparison to that of QDs without annealing process. Therefore, to obtain the best performance, the process temperature was chosen as 70 °C for InP/ZnS core/shell QD-LED fabrication.

**Fig. 5:**
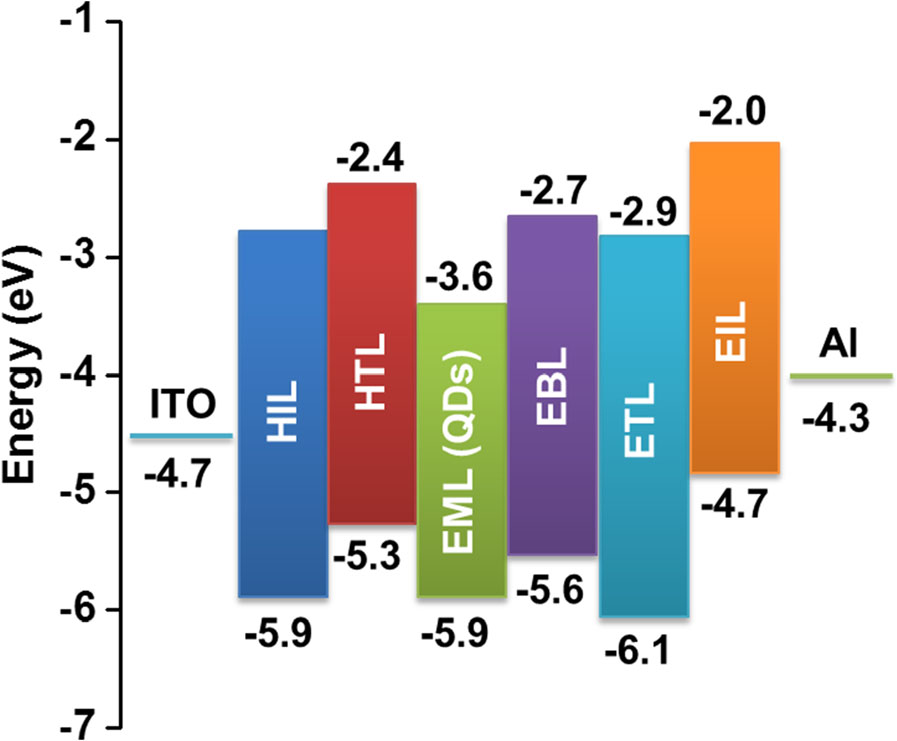
The energy levels of the individual layers of multilayered InP/ZnS core/shell QD-LEDs

**Fig. 6:**
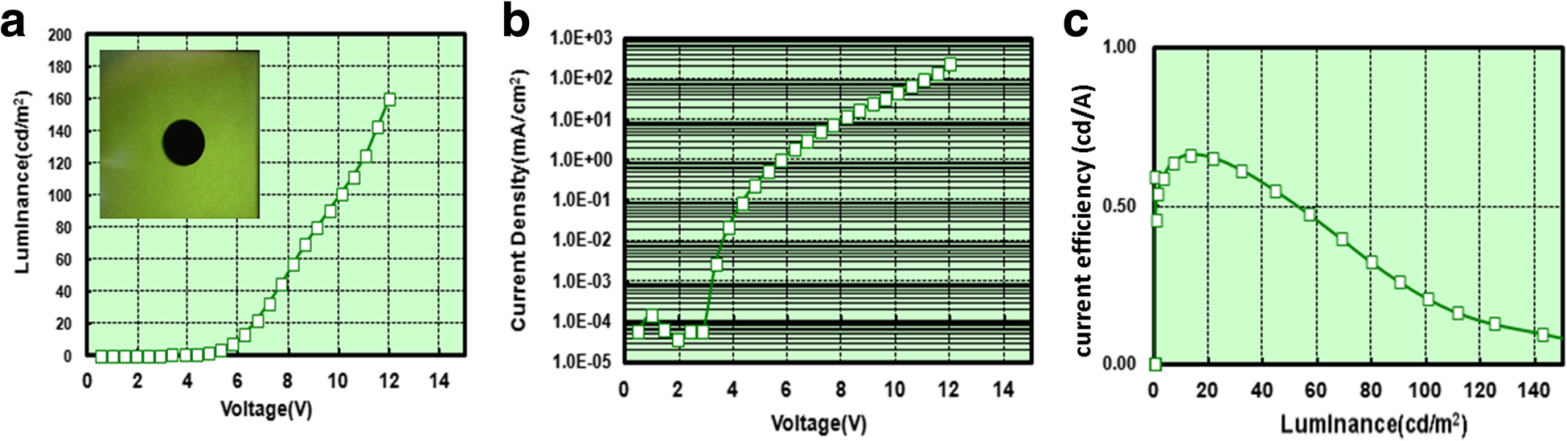
**a** Luminance-voltage characteristic. The inset shows the green multilayered InP/ZnS core/shell QD-LEDs. **b** Current density-voltage characteristic and **c** current efficiency as a function of luminance for multilayered InP/ZnS core/shell QD-LEDs

Multilayered InP/ZnS core/shell QD-LEDs were fabricated via sequential spin depositions of the constituent layers including HIL (30 nm), HTL (20 nm), InP/ZnS core/shell QDs (EML, 26 nm), EBL (10 nm), ETL (22 nm), and EIL (1 nm) on ITO glass substrate. Finally, a 150-nm-thick Al film was thermally deposited as a cathode. Figure 5 shows the energy levels of the individual layers of multilayered InP/ZnS core/shell QD-LEDs. Luminance-voltage characteristic of multilayered InP/ZnS core/shell QD-LEDs is presented in Fig. 6a. The turn-on voltage of multilayered InP/ZnS core/shell QD-LEDs was ~ 5 V. Furthermore, the multilayered InP/ZnS core/shell QD-LEDs showed the highest luminance (160 cd/m^2^) at 12 V. For the current density-voltage characteristic, the current of multilayered InP/ZnS core/shell QD-LEDs appeared at ~ 5 V and increased to 1.09 mA/m^2^ at 5.7 V as shown in Fig. 6b. The results indicated the efficient injection of holes and electrons into the InP/ZnS core/shell QDs layer. The current efficiency as a function of luminance for multilayered InP/ZnS core/shell QD-LEDs is shown in Fig. 6c. A maximum current efficiency of 0.65 cd/A and external quantum efficiency of 0.223% were achieved with multilayered InP/ZnS core/shell QD-LEDs at luminance ~ 20 cd/m^2^. Although the efficiency of multilayered InP/ZnS core/shell QD-LEDs is still not enough for the practical applications such as displays in this work, the development of QD-LEDs with environment-friendly materials, low cost, and high performance remains a key issue to make them more competitive for practical applications.

## Conclusions

Heavy-metal-free InP/ZnS core/shell QDs with different fluorescence were successfully prepared by solvothermal green synthesis with cheap, safer, and environment-friendly precursors including InI_3_, ZnCl_2_, (DMA)_3_P, zinc stearate, and sulfur compared to previous studies. The results of TEM characterizations showed that the InP/ZnS core/shell QDs with green fluorescence revealed the spherical morphology with the average diameter ~ 4 nm. The XRD pattern demonstrated the lattice mismatch of InP/ZnS core/shell QDs for core/shell structure. For the optical properties, the green fluorescent InP/ZnS core/shell QDs with superior fluorescence quantum yield of 60.1% and full width at half maximum of 55 nm were used as an emission layer to prepare multilayered QD-LEDs. The optimal process temperature was chosen as 70 °C for InP/ZnS core/shell QD-LED fabrication to obtain the best performance. The multilayered InP/ZnS core/shell QD-LEDs showed the turn-on voltage at ~ 5 V, the highest luminance (160 cd/m^2^) at 12 V, and the external quantum efficiency of 0.223% at 6.7 V. Although the multilayered InP/ZnS core/shell QD-LEDs were fabricated, the device long-term stability still remains a great challenge. The multilayered InP/ZnS core/shell QD-LEDs with low cost and environmental friendliness could be a potential candidate for future display applications.

## Additional file

Additional file 1: Calculation of fluorescence quantum yield. **Figure S1.** Histogram of size distribution of InP/ZnS core/shell QDs and Gaussian fitting. **Figure S2.** EDX analysis of InP/ZnS core/shell QDs. **Figure S3.** **a** UV-Vis spectra and fluorescence of InP/ZnS core/shell QDs with red fluorescence. **b** UV-Vis spectra and fluorescence of InP/ZnS core/shell QDs with yellow fluorescence. **c** The red (right) and yellow (left) fluorescence of InP/ZnS core/shell QDs with the irradiation by hand-held long-wave UV lamp. (DOC 559 kb)
