# Supplementary material for: Green Synthesis of InP/ZnS Core/Shell Quantum Dots for Application in Heavy-Metal-Free Light-Emitting Diodes
Source: Nanoscale Res Lett. 2017 Sep 19;12:537. doi: 10.1186/s11671-017-2307-2 (PMC5605476; doi:10.1186/s11671-017-2307-2)
Supplement: Additional file 1: — Calculation of fluorescence quantum yield. Figure S1. Histogram of size distribution of InP/ZnS core/shell QDs and Gaussian fitting. Figure S2. EDX analysis of InP/ZnS core/shell QDs. Figure S3. a UV-Vis spectra and fluorescence of InP/ZnS core/shell QDs with red fluorescence. b UV-Vis spectra and fluorescence of InP/ZnS core/shell QDs with yellow fluorescence. c The red (right) and yellow (left) fluorescence of InP/ZnS core/shell QDs with the irradiation by hand-held long-wave UV lamp. (DOC 559 kb) [file 11671_2017_2307_MOESM1_ESM.doc]

**Green Synthesis of InP/ZnS Core/Shell Quantum Dots for Application in Heavy-Metal Free Light-Emitting Diodes**

Tsung-Rong Kuo^1,2*^, Shih-Ting Hung^3^, Yen-Ting Lin^3^, Tzu-Lin Chou^3^, Ming-Cheng Kuo^4^, Ya-Pei Kuo^4^ and Chia-Chun Chen^3*^

^1^ Graduate Institute of Nanomedicine and Medical Engineering, College of Biomedical Engineering, Taipei Medical University, Taipei 11031, Taiwan

^2^ International Ph.D. Program in Biomedical Engineering, College of Biomedical Engineering, Taipei Medical University, Taipei 11031, Taiwan

^3^ Department of Chemistry, National Taiwan Normal University, Taipei 11677, Taiwan

^4^ AU Optronics Corporation, New Display Process Division, HsinChu 30078, Taiwan

Correspondence and requests for materials should be addressed to T.K. (email: trkuo@tmu.edu.tw) or to C.C. (email: cjchen@ntnu.edu.tw).

**Calculation of Fluorescence Quantum Yield**

Fluorescein in 0.1 M NaOH (QY = 0.95) was chosen as a standard reference. The fluorescence quantum yield of QDs in chloroform was calculated with the following equation:

$$QY=QY_{St}\frac{1-10^{-A_{St}}}{1-10^{-A}}\times\frac{\eta^{2}}{\eta_{St}^{2}}\times\frac{I}{I_{St}}$$

where *QY* is the fluorescence quantum yield of QDs, *QY_st_* is the fluorescence quantum yield of fluorescein, *A* is the absorbance of ODs at exited wavelength, *A_st_* is the absorbance of fluorescein at exited wavelength, *η* is the refractive index of chloroform (1.44), *η_st_* is the refractive index of water (1.33), *I* is the fluorescent intensity of QDs and *I_st_* is the fluorescent intensity of fluorescein.


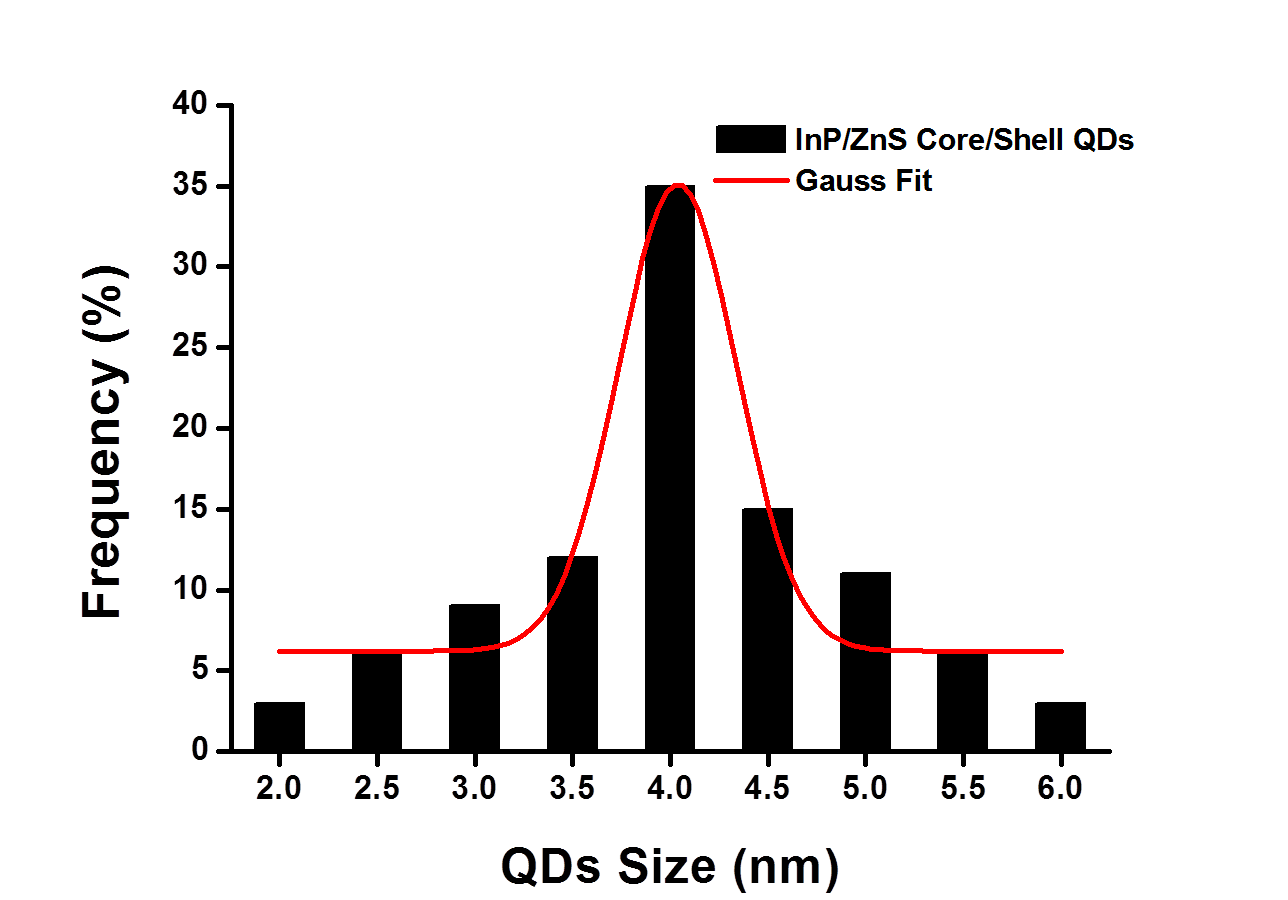


**Figure S1.** Histogram of size distribution of InP/ZnS core/shell QDs and Gaussian fitting.


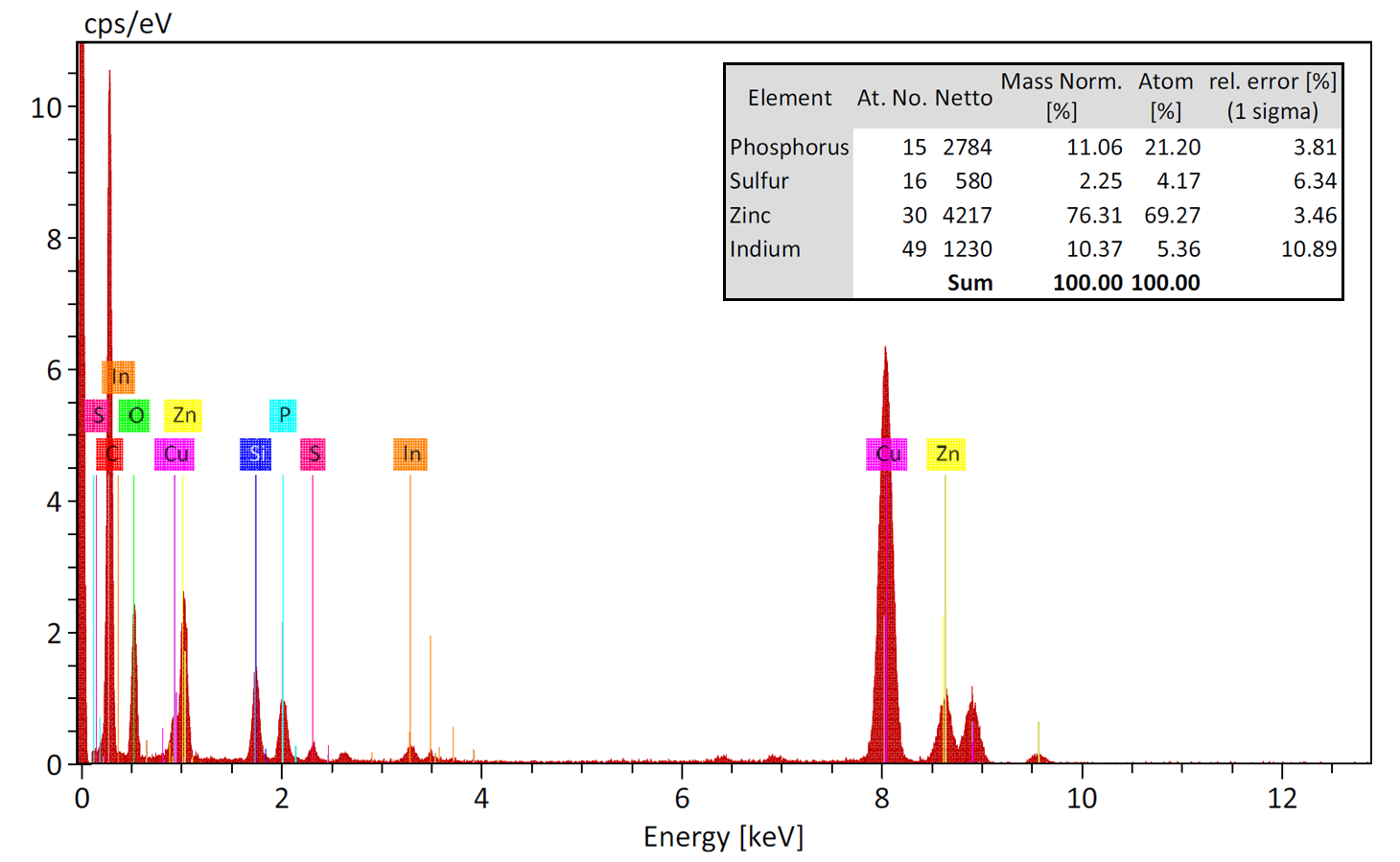


**Figure S2.** EDX analysis of InP/ZnS core/shell QDs.


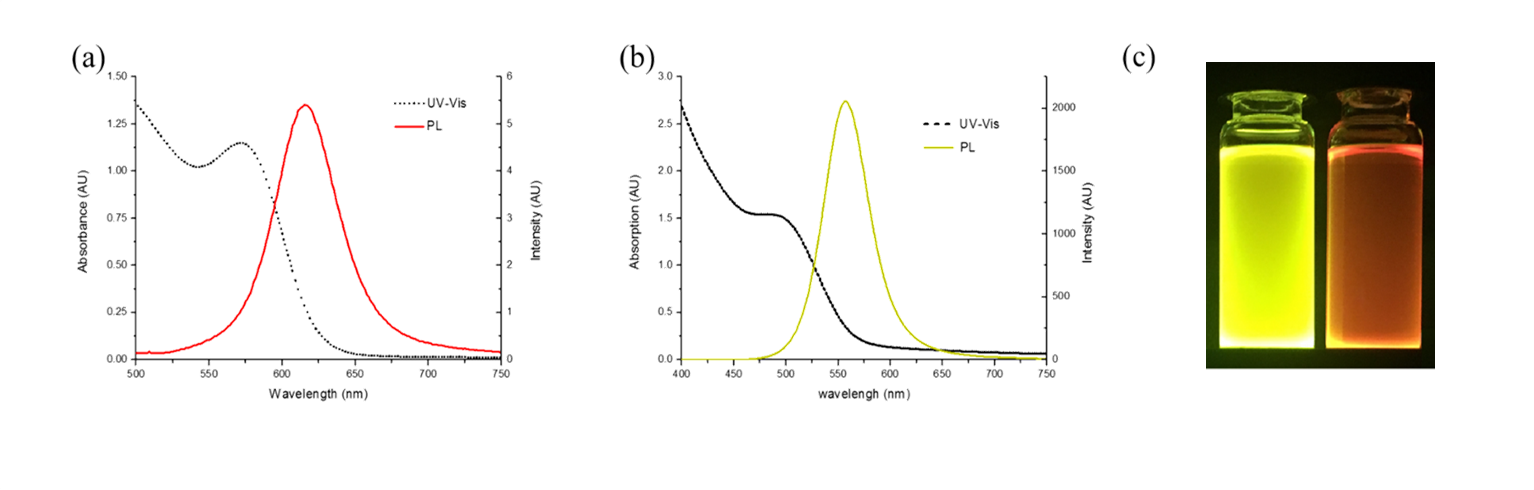


**Figure S3.** (a) UV-Vis spectra and fluorescence of InP/ZnS core/shell QDs with red fluorescence. (b) UV-Vis spectra and fluorescence of InP/ZnS core/shell QDs with yellow fluorescence. (c) The red (right) and yellow (left) fluorescence of InP/ZnS core/shell QDs with the irradiation by hand-held long-wave UV lamp.
